# Retroperitoneal Castlemans disease mimicking a liver cancer: a case report

**DOI:** 10.3389/fonc.2024.1343157

**Published:** 2024-02-12

**Authors:** Mao-Ji Kang, Jin-Chang Zhang, Cheng Fang, Bo Li, Song Su

**Affiliations:** Department of General Surgery (Hepatopancreatobiliary surgery), The Affiliated Hospital of Southwest Medical University, Sichuan, China

**Keywords:** Castleman disease, retroperitoneal, hepatic malignant tumor, contrast-enhanced computed tomographic, contrast-enhanced magnetic resonance imaging

## Abstract

Castleman disease (CD), a distinct lymphoproliferative disorder, is infrequently encountered in clinical practice and poses significant diagnostic challenges. We present the case of a 48-year-old asymptomatic female, admitted for evaluation of a hepatic mass detected in the liver’s right lobe. Preoperative laboratory tests were within normal limits. Diagnostic imaging, including contrast-enhanced magnetic resonance imaging (MRI), was suggestive of hepatocellular carcinoma. Furthermore, contrast-enhanced abdominal computed tomography (CT) scans were indicative of hepatic malignancy. Subsequently, the patient underwent laparoscopic surgery targeting a retroperitoneal mass. During the surgical procedure, it was observed that the tumor was a retroperitoneal mass situated posterior to the liver, exhibiting localized adhesion to hepatic tissue. The postoperative histopathological analysis revealed the mass to be hyaline-vascular type Castleman disease (HV-CD), thereby refuting the initial diagnosis of a hepatic malignancy. This case underscores the complexity of diagnosing retroperitoneal Castleman disease, particularly when it masquerades as a hepatic tumor.

## Introduction

Castleman disease (CD) is an uncommon form of reactive lymphadenopathy with an unknown etiology. It most commonly presents as a mediastinal nodal mass. CD can be divided into two categories based on clinical presentation and disease course: unicentric CD (UCD) and multicentric CD (MCD). The prognostic outcomes for the two subtypes of CD differ significantly. MCD typically exhibits a poor prognosis and is not amenable to curative surgical intervention, necessitating pharmacological management to control the disease. In contrast, UCD, often of the hyaline-vascular (HV) subtype, is usually amenable to curative resection. Approximately 80% to 90% of cases are classified histologically as hyaline vascular (HV-CD). HV-CD typically lacks systemic symptoms, and laboratory examination results and radiological information often lack specificity (1). Therefore, in previous reports, when the disease occurs in or resembles other organs such as the kidney, parotid gland, or pancreas, CD may be mistaken for other solid tumors (2–4). However, there are scant reports in the literature regarding cases of retroperitoneal CD masquerading as malignant hepatic tumors. Herein, we present a case of a 48-year-old female patient who was initially diagnosed with liver malignancy based on preoperative imaging but was subsequently confirmed to have retroperitoneal HV-CD through postoperative pathological examination. Six months following the procedure, the patient’s enhanced abdominal CT scan demonstrated favorable postoperative recovery with no evidence of disease recurrence.

## Case description

**Figure 1:**
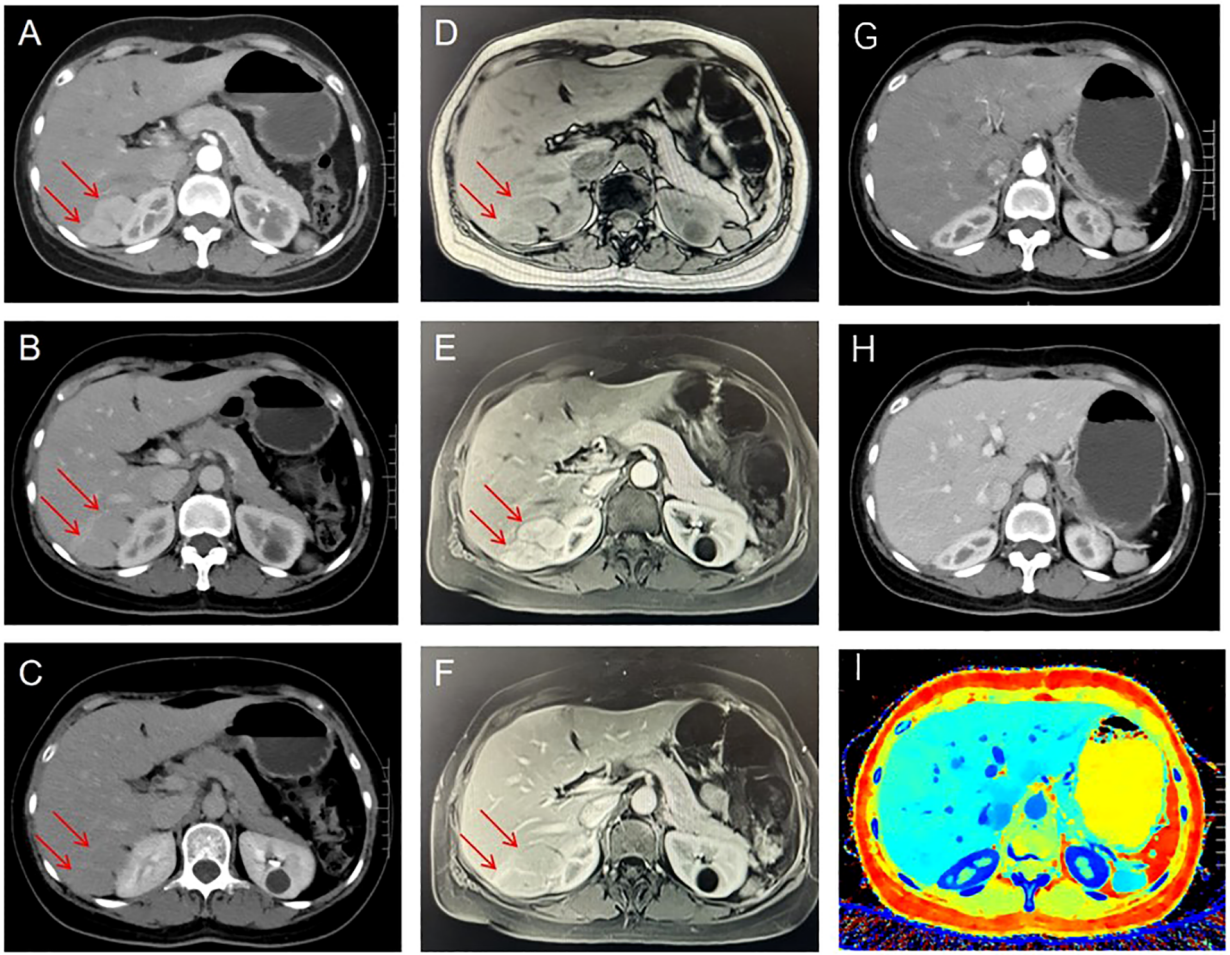
Preoperative contrast-enhanced CT showed a nodular and significantly enhanced lesion located in the right lobe of the liver during the arterial phase **(A)**, with reduced enhancement in the portal and delayed phases **(B, C)**. Preoperative Contrast-enhanced magnetic resonance imaging. Plain scan: T1W1 shows low signal intensity **(D)**, Enhanced scan: obvious enhancement in arterial phase **(E)**, less enhancement in portal phase and delayed phase than in surrounding liver parenchyma **(F)**. Six months after surgery, the results of contrast-enhanced CT examination did not reveal any notable abnormalities: arterial phase **(G)**; Venous phase **(H)**; Z Effective **(I)**. The tumor is indicated by red arrow.

A 48-year-old female patient was admitted to the hospital for further diagnosis and treatment following the discovery of a tumor in the right lobe of her liver during a health checkup one week earlier. The patient had no clinical manifestations. Contrast-enhanced magnetic resonance imaging (MRI) revealed a nodular mass in the right lobe of the liver with an enhancement pattern that appeared as fast-in and fast-out, leading to a diagnosis of hepatocellular carcinoma (**Figures 1D–F**). However, upon admission to the hospital, contrast-enhanced abdominal computed tomography (CT) revealed a nodular enhancement measuring approximately 3.1 cm × 2.0 cm in the right lobe of the liver at the arterial phase. The degree of strengthening at the portal vein stage and the delayed stage was reduced, indicating hepatic cancer (**Figures 1A–C**). Preoperative laboratory examinations, including tumor markers for the digestive tract, were normal.

**Figure 2:**
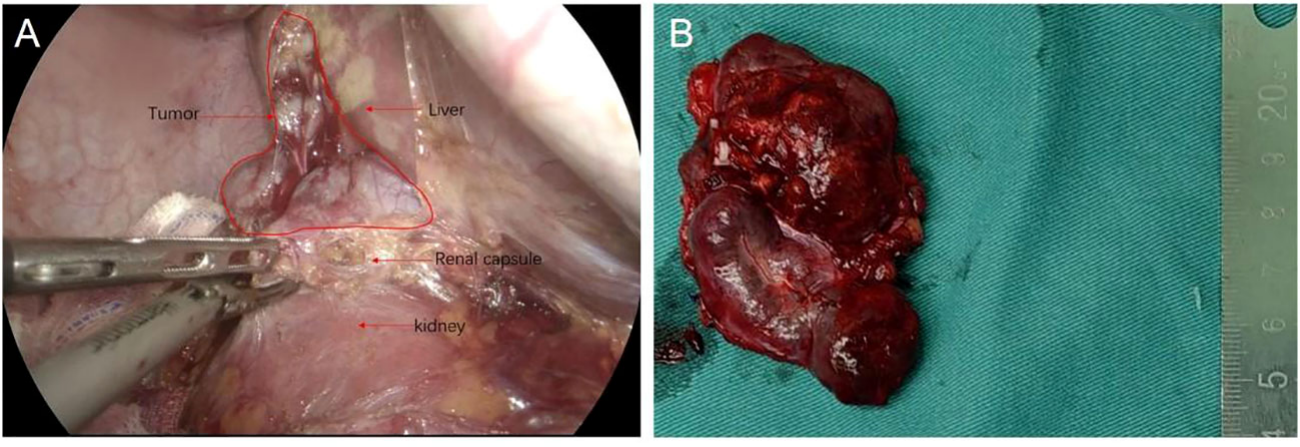
Intraoperative condition **(A)**. Completely resected tumor **(B)**.

After multi-disciplinary consultation, the tumor was considered most likely to be liver malignancy. Given the preoperative imaging findings suggesting tumor localization in segment VII of the liver, along with the inability to exclude a retroperitoneal origin of the tumor, a conventional laparoscopic right hepatectomy approach was proposed, designed to address both aforementioned scenarios and facilitate optimal exposure of the tumor. During the surgical intervention, an irregularly encapsulated tumor was identified adhering to the root of the right triangular ligament in the liver’s posterior lobe, in close proximity to the right renal capsule. (**Figure 2A**). The liver’s size and structure appeared normal. The adhesiolysis resulted in approximately 100 milliliters of hemorrhage. The tumor was completely resected and sent for pathological examination.

**Figure 3:**
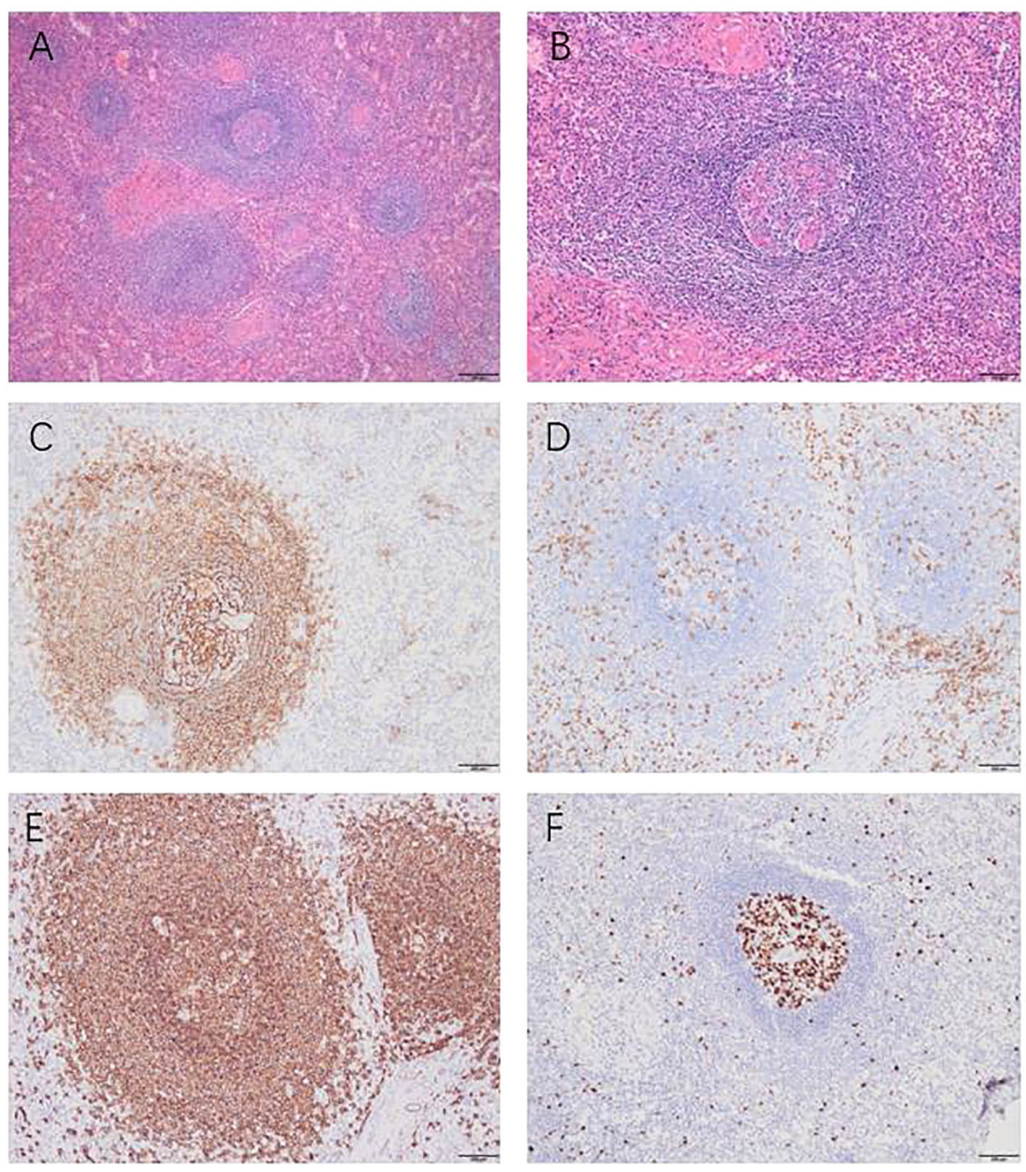
Histopathological and immunohistochemical findings, Histopathological examination **(A, B)**: “onion skin-like” structure. Immunohistochemical examination: CD21 was present in the concentric follicular dendritic cell networks **(C)**. CD3 was scattered positively among the T cells **(D)**. CD20 was scattered positively among the B cells **(E)**. KI67 showed high expression in the germinal center and low expression outside the germinal center **(F)**. Magnification x100 (panels A); x200 **(B–F)**.

Upon gross examination, the tumor appears grayish-red in color, irregular in shape, and measures approximately 7cm x 4.6cm x 2.1cm. Two nodules, ranging in diameter from 3.6cm to 4.2cm, are visible on the section. The nodules are solid and soft, with a complete capsule covering their surface, and they appear grayish-red upon sectioning (**Figure 2B**). Histopathological examination of the mass, the tumor exhibits an increased number of lymphoid follicles with an expanded mantle zone consisting of small lymphocytes and atrophic germinal centers, which gives rise to an “onion skin-like” structure. Additionally, there is vascular proliferation and hyalinization present in the interfollicular zones, as well as scattered follicular dendritic cells and plasma cells (**Figure 3**).

The immunohistochemical analysis conducted on the excised specimen revealed a distinctive pattern: CD20 and CD79ɑ were positively expressed among B cells, while CD3 and CD5 showed positive expression among T cells. In the germinal centers, there was positive expression of CD10 and Bcl-6, whereas Bcl-2 staining was notably absent. A marked elevation in KI67 expression was observed within the germinal centers, contrasting with lower expression levels in surrounding areas. Additionally, the specimen tested negative for IgG4 and CyclinD1 (**Figure 3**). Collectively, these immunohistochemical findings corroborate the diagnosis of HV-CD.

Postoperatively, the patient’s recovery was uneventful, with no adverse complications reported. One week following the surgical intervention, the patient demonstrated satisfactory recovery, warranting discharge from the hospital. A six-month postoperative follow-up involving an enhanced CT scan of the abdomen revealed no evidence of disease recurrence, confirming the effectiveness of the treatment (**Figures 1G–I**).

## Discussion

CD is a rare non-neoplastic lymphoproliferative disorder first described in 1954 by Castleman and Towne as a benign localized lump or mass. The disease has two subtypes: UCD and MCD. MCD is characterized by systemic inflammatory symptoms, organ system dysfunction, and laboratory abnormalities such as fever, weight loss, low protein levels, systemic lymphadenopathy, renal function damage, and sometimes the development of POEMS syndrome (polyneuropathy, organomegaly, endocrinopathy, protein and skin changes). In contrast, UCD has almost no clinical manifestation (5, 6). Lesions can appear anywhere in the body but are most commonly found in the mediastinum (7). This case details a retroperitoneal, asymptomatic UCD. The initial imaging findings closely mimicked those of malignant hepatic tumors. Ultimately, the lesion was successfully excised through localized surgical resection.

CD is often under-recognized due to its rarity, rendering the diagnostic process challenging. For a definitive diagnosis of UCD, lymphadenopathy must be limited to a single lymph node or a localized nodal region (1). This necessitates a comprehensive diagnostic work-up that includes not only histological analysis of an excised lymph node but also radiological evaluation, typically involving whole-body CT or FDG-PET. However, CT imaging often lacks distinctive features (8).

Existing literature documents instances of CD manifesting in the retroperitoneal or perirenal regions. Notably, Fu et al. (9) described a case of CD located posterior to the right lower abdominal peritoneum, presenting as an isolated heterogeneous soft tissue mass on CT scans. Additionally, S Okada et al. (10) reported on a perirenal retroperitoneal CD case, where T1-weighted MRI revealed signal intensity of the mass akin to muscle tissue, and T2-weighted MRI showed heterogeneous internal signal characteristics with peripheral enhancement. Diverging from these cases, the tumor in this case demonstrated rapid enhancement and washout patterns in both contrast-enhanced CT and MRI.

UCD typically demonstrates pronounced enhancement in the arterial phase on contrast-enhanced CT/MRI scans. Some cases may exhibit enhancement in the delayed phase, with visible blood supply vessels within or around the lesion being a common feature (8). Conversely, hepatic carcinoma characteristically presents with rapid enhancement and washout in contrast-enhanced imaging. In this case report, the preoperative imaging findings of the patient’s tumor bore similarities to those typical of hepatic carcinoma.

The metabolic activity in CD makes PET-CT a valuable diagnostic tool (11), though its high cost limits its widespread use. In a previous case report (12), UCD was accurately diagnosed preoperatively via endoscopic ultrasound-guided fine needle biopsy (EUS-FNB). In this case, a preoperative biopsy was not performed due to the high likelihood of a malignant hepatic tumor diagnosis and concerns about needle tract cancer metastasis. Additionally, the tumor’s deep location posed challenges for a safe and effective biopsy. Regardless of the hypothetical preoperative biopsy results, the tumor was amenable to complete surgical removal.

Histological examination is the gold standard for diagnosing CD, requiring lesion resection or biopsy. Most UCD cases undergo immediate surgical resection (13). To determine this CD case’s specific subtype and differentiate it from other differential diagnoses, including lymphoma and autoimmune disorders, additional immunohistochemical analyses were conducted. These findings also provide prognostic insights and guide therapeutic strategies for CD with varied immunophenotypes (14).

## Conclusion

We reported a rare case of UCD located in the retroperitoneum of the right hepato-renal space that was misdiagnosed as a liver malignant tumor due to the lack of clinical symptoms and imaging features. Paying attention to the patient’s history and conducting comprehensive comparisons of various types of imaging results may assist with the diagnosis. Preoperative biopsy also holds a pivotal role in the diagnostic process for this disease. CD has a good prognosis with surgical removal of the lesion. In cases where UCD is suspected, complete surgical resection may successfully treat the patient without an unnecessarily extensive resection.

## Data availability statement

The original contributions presented in the study are included in the article/supplementary material. Further inquiries can be directed to the corresponding author.

## Ethics statement

The studies involving humans were approved by Clinical Trial Ethics Committee of Southwest Medical University Affiliated Hospital. The studies were conducted in accordance with the local legislation and institutional requirements. The participants provided their written informed consent to participate in this study. Written informed consent was obtained from the individual(s) for the publication of any potentially identifiable images or data included in this article.

## Author contributions

M-JK: Data curation, Writing – original draft. J-CZ: Data curation, Writing – original draft. CF: Conceptualization, Writing – review & editing. BL: Conceptualization, Writing – review & editing. SS: Conceptualization, Writing – review & editing.
